# Impact of diabetes mellitus on the frequency of postoperative complications after carotid endarterectomy

**DOI:** 10.5830/CVJA-2022-054

**Published:** 2022-11-09

**Authors:** Gojko Lj Igrutinović, Aleksandar R Jakovljević, Nikola M Miljković, Mladen N Kasalović, Dragoslav Dj Nenezić, Zlatan N Elek, Danijela R Vićentijević

**Affiliations:** Surgery Clinic, Kosovska Mitrovica Clinical Hospital Centre, Kosovska, Mitrovica, Serbia; Institute for Cardiovascular Diseases, Dedinje, Belgrade, Serbia; Medical Faculty, Kosovska, Mitrovica, Serbia

**Keywords:** carotid endarterectomy, diabetes mellitus, post-operative complications

## Abstract

**Background:**

There is significant controversy surrounding the link between diabetes mellitus and post-operative complications after carotid endarterectomy (CEA). The aim of this study was to identify the possible effects of diabetes on the frequency of post-operative complications after CEA.

**Methods:**

This prospective study was conducted at the Dedinje Clinic for Vascular Surgery, Belgrade. The patients who underwent CEA were divided into two groups: group A (37.7%) included 98 (35.1%) insulin-dependent and 181 (64.9%) insulin-independent diabetic patients, and group B (62.3%) comprised non-diabetic subjects

**Results:**

The pre-operative characteristics were similar, except for a greater prevalence of dyslipidaemia in patients with diabetes. Post-operative cardiac events occurred more often in patients with diabetes (3.6%) than in non-diabetic patients (1.1%) (p = 0.039); post-operative neurological events among patients with diabetes were 3.6% and among non-diabetics, 0.9% (p = 0.009). Peri-operative mortality rate was 2.5% in the diabetic group and 0.9% in the non-diabetic group. The total percentage of post-operative complications was two or more times higher in the diabetic group than the non-diabetic group (8.5 vs 18.3%, p < 0.001).

**Conclusions:**

Diabetes mellitus increased the surgical risk of CEA. Higher rates of mortality and post-operative complications were observed in patients being treated with oral antidiabetics than in those on insulin.

Stenotic occlusive diseases of the carotid arteries are some of the most frequent surgical vascular diseases, and the incidence is increasing, and carotid endarterectomy (CEA) is the most frequent operation carried out at vascular surgery clinics.^1^ It is well known that diabetes mellitus (DM) is a progressive disease that significantly contributes to degradation of atherosclerotic plaques in the arteries.^2^

CEA represents the first choice of treatment of high-level carotid stenosis and carries a low rate of post-operative morbidity and mortality.^3^ According to the literature, the percentage of patients with DM within the group of patients who undergo CEA has been on the rise, ranging from 10% to over 25%.^4^,^5^ On the other hand, diabetes is an important risk factor for the occurrence of myocardial infarction (MI) and stroke in the general population, and it can also impact on the outcome of CEA.^6^

Some authors believe that patients suffering from DM are at up to two times greater risk of overall post-operative complications compared to patients with no diabetes.^6^-^8^ On the other hand, others have observed that patients suffering from DM were exposed to the same risk of cardiological morbidity, mortality and stroke after CEA as patients with no diabetes.^9^-^11^

It has been proven that there is a risk of stroke in diabetic patients and it is related to the level of hyperglycaemia; it is assumed that controlled glycaemia may lower the risk of stroke.^12^ Since most of these studies have not taken into consideration the type of antidiabetic therapy, this could explain the contradictory results among studies on the influence of DM on the outcome of CEA. The aim of this article was to determine the frequency of post-operative complications after CEA in patients with or without DM, as well as to identify the type of antidiabetic therapy, in order to define the safety of the operation concerned.

## Methods

This prospective, non-randomised study included all patients treated at the Institute for Cardiovascular Diseases, Dedinje, Belgrade, who, in the time period from December 2013 to December 2014, underwent CEA, and were entered into the prospective vascular registry. The patients were divided into two groups: group A (37.7%) consisted of 279 diabetic patients, including 98 (35.1%) being insulin-dependent and 181 (64.9%) taking oral hypoglycaemics. Group B (62.3%) consisted of 461 non-diabetics.

A complete comparison of demographic characteristics and risk factors was done (gender, age, hypertension, hyperlipidaemia, smoking, pre-operative MI, concomitant vascular diseases). After the operation we compared post-operative complications [transient ischaemic attack (TIA), cerebrovascular infarction (CVI), MI, neurological, cardiological and other morbidities, and total mortality rate].

Control colour duplex sonography (CDS) of the carotid artery was carried out one month, six months and one year later. Our primary goal was 30-day morbidity and mortality, which is defined as any death or post-operative morbidity within 30 days of the treatment, as well as a percentage of carotid restenosis up to one year post-operatively.

Indication for CEA was set after CDS of the carotid artery and multislice (MSCT) angiography of supra-aortic branches. Carotid stenosis was ascertained according to the European carotid surgery trial (ECST) criteria, as well as by means of the criteria described by AbuRahma et al.^13^ Carotid stenosis was defined as significant (> 70% constriction) if systolic velocity was > 150 cm/s and diastolic velocity > 90 cm/s.

Peri-operative neurological morbidity was classified as TIA lasting less than 24 hours or permanent stroke (deficiency present on discharge). An adverse post-operative cardiac event has been designated as a post-operative MI and congestive heart failure (CHF). Post-operative restenosis was defined as ultrasound-verified stenosis of the carotid artery on the operated side as larger than 50%.

Patients with a history of disease of the coronary artery underwent stress tests and, in some cases, coronarography. Peripheral vascular disease was proven by means of CDS of the lower extremities and, if deemed necessary, using angiography. For evaluation of pre-operative neurological disorders, as well as post-operative neurological condition, a modified scale of Rankin scores was used, with a neurological damage estimate ranging from 0 to 5.^14^

The patients were operated on under general endotracheal anaesthesia. The CEA eversion technique was applied on all patients. All patients with post-operative complications underwent a computed tomography (CT) of the endocranium post-operatively.

## Statistical analysis

The testing of statistical hypotheses made use of the t-test for two independent samples, Mann–Whitney test, chi-squared test and Fisher’s test of accurate probability. Logistic regression was used for analysing the relationship between binary outcomes and potential predictors. Statistical hypotheses were tested at the level of statistical significance (alpha level) of 0.05.

## Results

The main characteristics are shown in Table 1. Group A (37.7%) consisted of 279 diabetic patients and group B (62.3%) comprised 461 non-diabetic subjects. Except for a slightly higher prevalence of dyslipidaemia in patients with diabetes (χ2 = 5.330; p = 0.021), patients with DM had more frequent coronary artery disease (χ2 = 15.090; p < 0.001) and more persistent peripheral arterial disease (χ2 = 20.607; p < 0.001). Other pre-operative characteristics for the two groups were similar and comparable.

**Table 1:** Pre-operative characteristics of diabetics and non-diabetics

*Characteristics*	*Group A: diabetics 279 (37.7%)*	*Group B: non-diabetics 461 (62.3%)*	*p-value*
Average age	67.5 + 7.2	66.8 + 7.5	NS
Male, n (%)	165 (59.1)	278 (60.3)	NS
Female, n (%)	114 (40.9)	183 (39.7)	NS
Smoking, n (%)	169 (95.7)	257 (92.4)	NS
Hypertension, n (%)	267 (61.3)	426 (64.4)	NS
Dyslipidaemia, n (%)	265 (95.0)	416 (90.2)	0.021
Concomitant coronary disease, n (%)	89 (31.9)	89 (19.3)	< 0.001
Concomitant peripheral disease, n (%)	70 (25.1)	56 (12.1)	< 0.001
Previous MI, n (%)	38 (13.6)	30 (6.5)	0.001
Previous TIA, n (%)	10 (3.6)	4 (0.9)	NS
Previous CVI, n (%)	9 (3.2)	6 (1.3)	NS
Positive CT of endocranium	12 (4.66)	10 (2.17)	NS

MI, myocardial infarction; TIA, transient ischaemic attack; CVI, cerebrovascular

incident.

Post-operative complications are shown in Table 2. Neurological events (TIA) among patients with diabetes were 3.6% and among non-diabetics (0.9%) (p = 0.009). Post-operative CVI occurred in 1.3% of patients without diabetes and in 3.2% of patients with diabetes (χ2 = 3.241; p = 0.072). Cumulative neurological events TIA/cerebrovascular infarction were also statistically more numerous in the diabetic group (p = 0.02).

**Table 2:** Post-operative complications in diabetics and non-diabetics after CEA

*Characteristics*	*Group A: diabetics*	*Group B: non-diabetics*	*p-value*
Post-operative TIA, n (%)	10 (3.6)	4 (0.9)	0.009
Post-operative CVI, n (%)	9 (3.2)	6 (1.3)	0.072
CT ischaemic brain lesion, n (%)	11 (3.94)	8 (1.74)	0.424
Cranial nerves lesion, n (%)	2 (0.7)	4 (0.9)	NS
Myocardial infarction, n (%)	2 (0.7)	1 (0.2)	0.300
Congestive heart failure, n (%)	8 (2.9)	4 (0.9)	0.039
Post-operative respiratory complications, n (%)	8 (2.9)	3 (0.7)	0.024
Haematoma of operated wound, n (%)	10 (3.9)	11 (2.4)	0.341
Infection of operated wound, n (%)	5 (1.8)	0 (0.0)	0.007
Post-operative 50% restenosis, n (%)	5 (1.8)	10 (2.2)	0.724

TIA, transient ischaemic attack; CVI, cerebrovascular incident.

**Table 3:** Total mortality and morbidity rates in patients with diabetes and without diabetes who underwent CEA

*Structure of patients*	*Group A: diabetics*	*Group B: non-diabetics*	*Total*	*p-value*
Fatal cardiac event, n (%)	4 (1.4)	3 (0.7)	7 (0.9)	0.435
Fatal neurological event, n (%)	3 (1.1)	1 (0.2)	4 (0.5)	0.153
Total mortality, n (%)	7 (2.5)	4 (0.9)	11 (1.5)	0.113
Total morbidity, n (%)	51 (18.3)	39 (8.5)	90 (12.2)	< 0.01

Adverse post-operative cardiac events (MI, CHF) occurred in 3.6% of patients with diabetes and in 1.1% of non-diabetic patients (Fisher’s test of accurate probability; p = 0.039).

Haematoma of the surgical wound occurred in 11 patients (2.4%) without DM and in 10 patients (3.6%) with DM, which was statistically significantly different (χ2 = 0.905; p = 0.341). Infection of the operated wound in our study was present in 1.8% of patients with DM, while none of the patients without DM had wound infection, which was statistically significant (Fisher’s test of accurate probability; p = 0.007).

We observed that 0.9% of non-DM patients and 0.7% of DM patients had symptoms of cranial nerve lesions, which was statistically insignificant (Fisher’s test of accurate probability; p = 1.000). Post-operative restenosis occurred in 2.2% of patients without DM and in 1.8% of those with DM.

The total rate of complications, shown in Table 3, was within the recommended limits. With regard to the rates of mortality and total morbidity, the two groups differed considerably from one another. Operative and post-operative mortality (neurological and cardiological) was 2.5% in the diabetic group (four cardiac events and three cerebrovascular infarctions) and 0.9% in the non-diabetic group (three MIs and one cerebrovascular infarction) (p = 0.113). The patients with DM had statistically significantly higher total mortality rates.

Total post-operative complications were observed in 8.5% of patients without DM and in 18.3% of patients with DM (χ2 = 15.688; p < 0.001). Post-operative complications occurred in DM patients considerably more frequently (two or more times).

One hundred and eighty-one DM patients (64.9%) were using oral antidiabetics and 98 (35.1%) were on insulin. Total postoperative complications occurred in 25.4% of patients on oral antidiabetics and in 8.2% of patients on insulin (χ2 = 12.122; p < 0.001). Post-operative complications occurred in patients on oral antidiabetics considerably more frequently than in those using insulin (Table 4).

**Table 4:** Distribution of patients depending on diabetes therapy, in relation to post-operative complications

	*antidiabetics*	*Oral*	*Insulin*	*therapy*		*Total*
*Post-operative complications*	*n*	*%*	*n*	*%*	*n*	*%*
Not present	135	74.6	90	91.8	225	80.6
Present	46	25.4	8	8.2	54	19.4
Total	181	100.0	98	100.0	279	100.0

The stratification of patients with diabetes according to their levels of glycosylated haemoglobin (HbA1c) has shown that the group with HbA1c levels > 7.6 % had a higher total morbidity and mortality rate (Table 5). The median value of HbA1c in patients without post-operative complications was 7.6 ± 1.2, whereas the median value of HbA1c in patients with post-operative complications was 8.4 ± 0.9 (t = 5.010; p < 0.001). Patients with post-operative complications had significantly higher values of HbA1c.

**Table 5:** HbA1c level in examined patients

*HbA 1c level*	*Number*	*%*	*SD*	*Median*	*Minimum*	*Maximum*
Without post-operative complications	225	7.6	1.2	8.0	6.0	9.8
With post-operative complications	54	8.4	0.9	8.5	6.0	9.5
Total	279	7.8	1.2	8.0	6.0	9.8

The multiple logistic regression model, with post-operative complications as a dependent variable, was supplemented with those predictors of post-operative complications that were statistically significant in the simple logistic regression model, the significance level being 0.05. Statistically significant predictors of early post-operative complications in the multiple logistic regression model were: age (B = 0.069; p < 0.001) with odds ratio (OR) = 1.07, and it demonstrates that with increase in age of one year there is a 7% greater risk for patients to develop early post-operative complications. Diabetes (B = 0.854; p = 0.001) had an OR of 2.35, showing that diabetic patients were, with all other factors in the model controlled, at 2.35 times greater risk of the development of early post-operative complications. Concomitant coronary artery disease patients (B = 0.844; p = 0.001), (OR = 2.33) were at 2.33 times greater risk of the development of post-operative complications, with all other factors in the model controlled.

With logistic regression, the factors identified to increase the odds of death and post-operative complications were: hyperlipoproteinaemia (p = 0.021), more persistent coronary artery disease (p = 0.001) and a higher frequency of peripheral arterial disease (p < 0.001). The factors determined to increase the odds of death and total morbidity were: higher levels of HbA1c (p < 0.001) and oral antidiabetics for controlling glucose levels (p < 0.001), which is shown in Tables 4 and 5.

## Discussion

Although previous studies evaluated the connection between diabetes and a greater operative risk during CEA, there are various conflicting results in many studies. Most studies introduce CEA as a well-known and permanent procedure for the prevention of TIA and cerebrovascular infarction in patients with significant stenosis of the carotid artery.^15^

In previous studies, which included patients operated on for stenotic occlusive disease of the carotid arteries, the percentage of diabetics in most cases ranged from 13 to 23.6%.^6^,^15^ Our series included 37.7% of patients with diabetes, which is considerably higher compared to the study done by Ahari et al., which had 13% diabetic patients.^5^ Dorigo et al. report that the percentage of DM patients was 20.05%, whereas the study by Rockman et al. reports 23.5%.^6^,^9^ The study done by Jeong et al. reports on a high percentage of diabetics in Asia, up to 39%.^16^ The high percentage of diabetics in our study can be explained by the fact that DM has been reaching epidemic proportions in the general population over the last two decades, and especially in the group of patients with atherosclerotic disease.

Our study shows that pre-operative factors believed to increase the risk of death and stroke in diabetics include higher low-density lipoprotein values, coronary artery disease and peripheral arterial disease. A prompt diagnosis of these co-morbidities and the use of statins should reduce mortality and stroke rates after CEA.

Dorigo and co-workers state that patients with diabetes were predominantly women, who suffered from coronary artery disease, peripheral arterial disease and hyperlipidaemia.^6^ Other authors claim that pre-CEA diagnosed risk factors such as atherosclerosis and diabetes had an effect on 30-day mortality rate and stroke, but they did not record a significant influence from dyslipidaemia.^17^

Our research, however, has identified most frequent postoperative complications, such as post-operative MI, coronary insufficiency, TIA, CVI, respiratory insufficiency, post-operative bleeding and wound infection. All these complications were significantly more prevalent in patients with DM.

Post-operative TIA was present in 0.9% of patients without DM and in 3.6% of patients with DM (p = 0.009), while postoperative CVI was 1.3 versus 3.2%, respectively (p = 0.072).

Patients with DM suffer significantly more often from early post-operative TIA and CVI. The greater risk of cerebrovascular infarction in patients with DM was reported in the recent review by Hussain et al., wherein they determined that DM was associated with a 1.5-times greater risk of stroke after CEA.^18^

Contrary to these statements, Ballota et al. suggest that there is no important difference in the frequency of these post-operative complications between diabetics and non-diabetics.^10^

Post-operative cardiological complications, including MI, occurred in 1.1% of patients without DM and in 3.6% of patients with DM (p = 0.039). However, that can be explained by the fact that our study pool consisted of a group of diabetics whose pre-operative cardiological complications were generally more prevalent. Altinbas and co-workers suggest no significant difference in post-operative cardiac events, and noted that after CEA a consequent coronary insufficiency occurred in 2.3% of cases, with no difference observed between the groups concerned.^19^ Ombrellaro et al. recently observed in post-CEA patients undesirable cardiac events such as MI and CHF in 14.3% of patients with diabetes and in 16% of the non-diabetic group. The difference was not statistically significant.^20^

The frequency of haematoma of the operated wound occurred in 2.4% of non-DM patients and in 3.6% of DM patients, while wound infection was present in only patients with DM (1.8%). Zhao et al. stated that the incidence of haematoma was relatively high in diabetics, the cause being pre-operative high doses of heparin (1 mg/kg), as well as double antiaggregation therapy.^17^ The same authors specify that diabetes may increase the possibility of wound and systemic infection, and that pre-operative HbA1c levels should be about 7% to reduce possible infections.^17^

Post-operatively, 50% carotid restenosis in the course of one year was not significant; it occurred in 2.2% of non-DM patients and in 1.8% of DM patients (χ2 = 0.124; p = 0.724). Other investigations produced similar results.^19^

Total post-operative mortality (neurological and cardiological) was present in 0.9% of non-diabetics and in 2.5% of diabetics (p = 0.113). Ahari et al. report in their study that diabetics had higher 30-day mortality rates (3.2 vs 1.4%; p = 0.02).^5^

Total post-operative complications were observed in 8.5% of non-diabetics and in 18.3% of DM patients (p < 0.001). DM patients were at more than two times greater risk of suffering from post-operative complications. Dorigo et al. found that the risk of post-operative complications was twice as high in patients suffering from DM.^6^ The study by Jeong et al. concluded that DM patients were not at a greater risk of 30-day morbidity and mortality after CEA than those without DM.^16^

Our patients on oral antidiabetics suffered considerably more often from post-operative complications than those on insulin (25.4 vs 8.2%; p < 0.001). In the study by Axelrod et al. there was a somewhat higher percentage of post-operative complications in the diabetics, although without a significant difference between the patients on insulin and those on oral antidiabetics.^21^ Dimić et al. have shown the cumulative rate of TIA/cerebrovascular infarction (p = 0.02) to be greater in insulin-dependent diabetics (IDDM) than in those who are insulin independent (IIDM).^22^

Similarly, Bennett et al. stated that insulin-necessitated DM is one of the independent predictors of high morbidity and mortality rates among the patients who have undergone CEA.^23^ However, Dorigo and co-workers discovered that patients with diabetes were at greater risk of death, but with no difference between the patients with insulin-controlled diabetes and those on oral medication.^6^ Parlani and colleagues reported that patients with IDDM had higher rates of cerebrovascular infarction and death (6.5 vs 1.7%; p = 0.02) than non-diabetics.^7^ Comparably, Pothof et al. stated that patients with IDDM had higher rates of 30-day cerebrovascular infarction and death than those without diabetes (3.4 vs 1.5%; p < 0.001).^24^

In our series, a more significant rate of mortality and post-operative complications occurred in diabetics being treated with oral antidiabetics, compared to those being treated with insulin, which conflicts with other studies that reported a higher frequency of complications in groups on insulin therapy. To some extent, this can be explained by extremely lengthy and irregular therapy of patients on oral antidiabetic agents, which leads to chronic, atherosclerotic changes in the blood vessels. Patients on insulin may be more diligent in their therapy.

With regard to long-term regulation of glycaemia, our patients with post-operative complications had significantly higher values of HbA1c (t = 5.010; p < 0.001). Tanashian et al. reported that the presence of DM was associated with an increased risk of ischaemic lesions in the brain and a higher percentage of post-operative complications, associated with increased values of glycaemia (8.0 mmol/l) and HbA1c (7.8–8%) in the pre-operative phase.^25^ Dimić et al. stated that the group of diabetics with HbA1c > 7% had a greater cumulative rate of TIA/ cerebrovascular infarctions (p = 0.03).22 Parr et al. reported that patients with IDDM, when compared to those with IIDM, had higher rates of cerebrovascular infarction (3.27, 0.93 and 0.94%; p < 0.0001), MI (3.35, 1.10 and 0.87%, p < 0.0001) and hospital mortality (p < 0.0001).26 Jeong et al. have shown that insulin use was associated with a higher rate of mortality and morbidity. The absence of data on serial measurements of HbA1c levels in their analysis was the reason they could not explain differences in glycaemic control.^16^

It is certain that high concentrations of low-density lipoprotein and chronic hyperglycaemia, indicated by high HbA1c levels, increases the development of atherosclerosis, which sets in earlier and is more pervasive in diabetics. On the basis of the results of our investigation, it is believed that the percentage of post-operative complications may be reduced by means of a better regulation of glycaemia, lower values of HbA1c, prompt diagnosis of glucose intolerance and regular and adequate antidiabetic therapy. Considering the small number of studies that have dealt with this kind of investigation of complications related to diabetes therapy, the hypothesis remains to be proven in similar future randomised studies.

## Conclusions

In comparison to other studies, this research, possibly for the first time, included a large number of patients for a short time period, a high percentage of diabetics were included, the investigation was conducted in diabetics on different types of antidiabetic therapy, and the occurrence of complications was determined according to values of glycosylated haemoglobin.

The results of this study indicate that diabetes was an independent risk factor for fatal and non-fatal cardiac or neurological events after CEA since it caused 2.5 times more post-operative complications in the group of diabetic patients. Our study also recorded a higher rate of mortality and post-operative complications, as well as higher HbA1c values in the diabetics on oral antidiabetics than in those on insulin therapy. This was in conflict with similar studies. Ultimately, these results show that CEA was a reliable and efficient method of surgical treatment of the patients with significant carotid stenosis and concomitant diabetes mellitus, regardless of the type of antidiabetic therapy. 
